# Humanising the mouse genome piece by piece

**DOI:** 10.1038/s41467-019-09716-7

**Published:** 2019-04-23

**Authors:** Fei Zhu, Remya R. Nair, Elizabeth M. C. Fisher, Thomas J. Cunningham

**Affiliations:** 1 Department of Neuromuscular Diseases, Institute of Neurology, University College London, London, WC1N 3BG UK; 2 Mammalian Genetics Unit, MRC Harwell Institute, Oxfordshire, OX11 0RD UK

**Keywords:** Genetic engineering, Disease genetics, CRISPR-Cas systems, Experimental models of disease

## Abstract

To better understand human health and disease, researchers create a wide variety of mouse models that carry human DNA. With recent advances in genome engineering, the targeted replacement of mouse genomic regions with orthologous human sequences has become increasingly viable, ranging from finely tuned humanisation of individual nucleotides and amino acids to the incorporation of many megabases of human DNA. Here, we examine emerging technologies for targeted genomic humanisation, we review the spectrum of existing genomically humanised mouse models and the insights such models have provided, and consider the lessons learned for designing such models in the future.

## Abstract (web summary)

Generation of transgenic mice has become routine in studying gene function and disease mechanisms, but often this is not enough to fully understand human biology. Here, the authors review the current state of the art of targeted genomic humanisation strategies and their advantages over classic approaches.

## Introduction

Genetically modified mice facilitate the analysis of gene function and regulation, as well as the dissection of disease mechanisms. Transgenic mice carrying human complementary DNAs (cDNAs) or mini-genes at non-endogenous loci have been around for decades (herein called ‘classic transgenics’)^1^, as have gene-targeted mice in which changes are made to specific DNA sequences in the mouse genome. Researchers increasingly combine these approaches and turn to the targeted genomic humanisation of mice, creating ‘knock-in’ mouse models in which a mouse sequence is replaced by the human orthologous DNA^2^. Compared with the thousands of classic transgenic mouse strains that mostly carry human DNA as randomly inserted, multicopy transgenes, the number of targeted genomically humanised mouse strains is low (Tables 1 and 2). Such mice are designed to have greater physiological relevance than their classic transgenic counterparts, which includes maintaining the correct genomic context of a gene of interest to preserve physiological expression levels and correct spatiotemporal expression patterns. Where non-coding human sequences are incorporated, human-specific regulation and human gene splice isoforms may be maintained. In addition, the translated protein is expected to display the unique biochemical properties of the human gene, including potentially unique deleterious properties when mutated.

**Table 1:** Examples of partial and smaller scale genomically humanised mice created for different investigations

Human gene/locus	Detail	Human KI size	Technology	References
Partial humanisation				
*IGK constant region*	Antibodies with human immunoglobulin kappa (light chain) constant regions	0.5 kb	HR in ES cells	^126^
*IGHG1 constant region*	Antibodies with human immunoglobulin gamma-1 (heavy chain) constant regions	2.1 kb	HR in ES cells	^127^
*FOXP2*	Humanising 2 human-specific residues (related to human speech)	2 bp	HR in ES cells	^13^
*APP*	Humanising 3 residues in Aβ domain plus human mutations (Alzheimer's disease)	3–7 bp	HR in ES cells	^19– 21^
*APOE*	Humanising 1 residue critical to human APOE4 biochemistry (Alzheimer's and cardiovascular disease)	1 bp	HR in ES cells	^26^
*TP53* (*p53*)	Humanising core DNA-binding domain plus human mutations (cancer)	2.8 kb	HR in ES cells	^30– 32^
*BDNF*	Modelling 2 human variant residues (psychiatric disorders)	274 bp	HR in ES cells	^33^
*OPRM1*	Humanising exon 1, with one of two human polymorphic variants (alcoholism)	0.7 kb	HR in ES cells	^35^
*AR*	Humanising exon 1 including Q-tract expansions (spinal bulbar muscular atrophy)	1.6 kb	HR in ES cells	^75, 81, 82^
*HTT*	Humanising exon 1 including Q-tract expansions (Huntington disease)	250–500 bp	HR in ES cells	^83– 92^
*TNFSF11*	Humanising single exon; target of monoclonal antibody drug (bone disorders)	0.4 kb	HR in ES cells	^37^
*FUS*	Recapitulating FUS splice site mutation (‘delta14’), plus human frameshifted C-terminus (ALS)	1 bp + 9 bp	HR in ES cells	^8^
*IKAP*	Humanising exon 20 + flanking introns, including human mutation (familial dysautonomia)	1.5 kb	HR in ES cells	^38^
*FKTN*	Humanising exon 10 ± human-specific SVA retrotransposon insertion (Fukuyama muscular dystrophy)	6 + 3 kb	RMCE in ES cells	^93^
*PRNP*	Humanising prion protein gene with mutation, minus signal peptide (prion diseases)	0.8 kb	HR in ES cells	^103^
*CTLA4*	Humanising gene minus signal peptide, to study anti-human CTLA4 antibody efficacy (cancer)	3.2 kb	HR in ES cells	^104^
*DMPK*	Humanising exons 13–15 plus CTG repeats (myotonic dystrophy)	1.5–1.7 kb	HR in ES cells	^105^
Humanising non-coding variants				
eQTL rs2277862; T	To model lipid-functional non-coding human variant	5 bp	CRISPR/Cas9-assisted HR in zygotes	^122^

All mice are cited in the text

**Table 2:** Examples of whole gene and larger-scale genomically humanised mice created for different investigations

Human gene/locus	Detail	Human KI size	Technology	References
Whole gene humanisation				
APOE	APO-E2, -E3, -E4 human variants (Alzheimer's and cardiovascular disease)	4.1 kb	HR in ES cells	^39– 41^
*IL3, TPO, CSF1, CSF2, IL15, SIRPA*	Supporting the human cellular component of chimaeric animals with a human immune system in immunodeficient mice	8.5–17.5 kb	HR in ES cells	^44– 48^
*C5AR1*	To generate and study anti-human C5AR1 antibody efficacy (inflammation)	1 kb	HR in ES cells	^49^
PXR, CAR	Xenobiotic sensors; predicting human drug responses	4.3 kb and 7 kb	HR in ES cells	^52, 53^
*GCGR*	Humanising target of monoclonal antibody drug (diabetes)	4.7 kb	HR in ES cells	^62^
*SIRPA* and *CYP2D6*	Testing ssODN-mediated end joining	199.5 and 6.2 kb	ssODN-mediated end joining in rat zygotes	^124^
*KMT2D*	Human cancer gene KI; testing CRISPR/Cas9-assisted HR in ES cells and homology arm lengths	42 kb	CRISPR/Cas9-assisted HR in ES cells	^128^
RHO	Whole Rhodopsin gene plus GFP tag (retinal degeneration)	7.4 kb	Both HR and RMCE in ES cells	^106^
Humanising large loci and gene clusters				
*IGH* and *IGK* variable regions	Antibodies with human variable regions	2.6 + 3 Mb	Iterative HR in ES cells	^56, 57^
*IGH, IGK,* and *IGL* variable regions	Antibodies with human variable regions	﻿917 + 838 + 932 kb	S-RMCE in ES cells	^55^
* α globin* cluster	Proof-of-principle for large humanisation and to study α globin gene expression	117 kb	RMGR	^58^
β *globin* genes	To study β thalassaemia	8.7–11.7 kb	HR in ES cells	^94, 97– 99^
*CYP3A4/CYP3A7*	To study cytochrome P450-mediated drug metabolism	~100 kb	RMCE in ES cells	^51^
Transchromosomic mice				
Chromosome 21	Transchromosomic mouse model for Down syndrome	42 Mb	MMCT	^54, 60, 61, 70, 71, 107^
Immunoglobulin loci transchromosomic mice	Transchromosomic mouse model for human monoclonal antibody production	1.5 + 2 Mb	MMCT	^102, 129^

All mice are cited in the text

Genomically humanised mouse strains with physiological levels of gene expression tend to have slower, milder phenotypes than transgenic overexpression models. However, these gene-targeted animals avoid overexpression artefacts, ectopic expression, and mutations resulting from random integration^3,4^ that can arise in transgenic models. Endogenous expression levels are particularly important when modelling dosage-sensitive genes, such as the RNA binding proteins *C9orf72*^5^, *TARDBP* (Tar DNA-binding protein that encodes TDP-43)^6,7^ or *FUS* (fused in sarcoma)^8,9^. Mutations in these genes can be causative for the neurodegenerative disease amyotrophic lateral sclerosis (ALS), which is characterised by progressive motor neuron loss and paralysis. In contrast, overexpression of the human wild-type FUS alone is sufficient to cause motor neuron degeneration and other deficits in transgenic mice, thereby limiting the study of pathogenic mutations^10^ and making it necessary to tightly control gene dosage in these animal models.

Thus, genomically humanised mice offer refined models of human biology and pathology, as well as models for testing small molecule drug therapies, antibody therapeutics^11^, and gene therapies such as antisense oligonucleotides^12^, as we will discuss below.

**Fig. 1:**
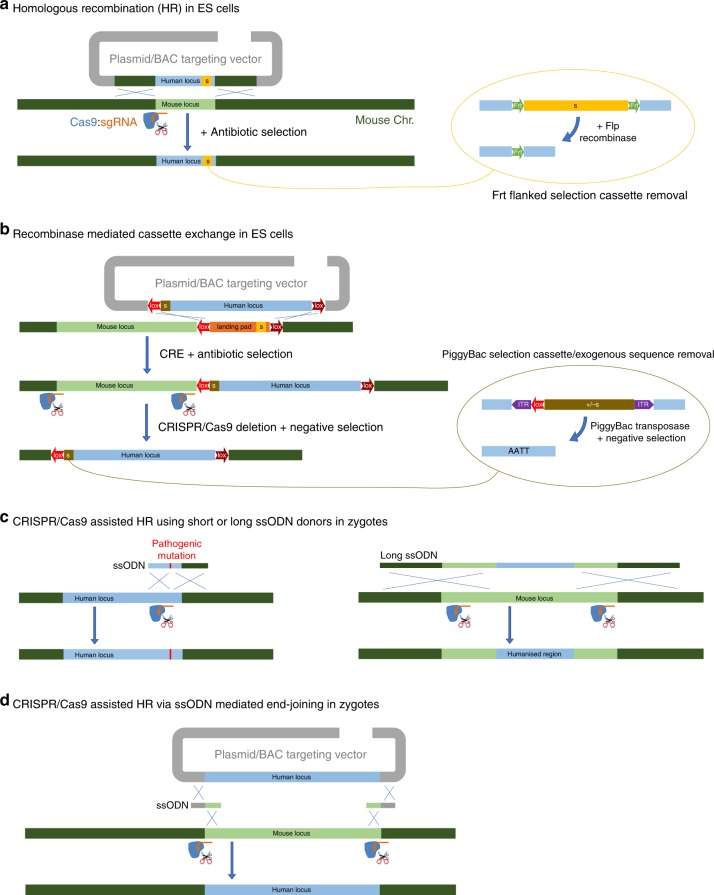
Targeted genomic humanisation technologies. **a** HR in ES cells has been used to humanise loci up to ~200 kb (and beyond, using iterative targeting). A plasmid, or BAC, targeting vector carrying human sequence flanked by homology arms is transfected into ES cells by electroporation. Addition of Cas9:sgRNA, generating a targeted double strand break, increases HR efficiency. An antibiotic resistance selectable marker is included to enrich for ES cells harbouring the desired recombination. Selection cassettes are commonly flanked by frt sites for later excision by FLP recombinase, leaving a single frt genomic scar. **b** Recombinase-mediated cassette exchange (RMCE) can be used to humanise up to ~200 kb loci (can also be employed iteratively). In this example, a landing pad is first inserted at the target locus via HR (see part **a**), consisting of a selection cassette flanked by heterotypic lox sites. The same lox sites are inserted either side of the orthologous human locus within a BAC vector, which when electroporated into landing pad-harbouring ES cells will recombine in the presence of CRE recombinase. Cas9:sgRNA pairs can subsequently be utilised to delete the mouse locus. As an alternative to FLP/frt recombination, selection cassettes and other exogenous sequences can be flanked by PiggyBac inverted terminal repeats (ITR), which when inserted at an AATT recognition site, leave no genomic scar once excised with PiggyBac transposase. PiggyBAC transposition is less efficient than FLP/frt recombination, thus positive–negative selection cassettes (+/− s) such as HPRT (in HPRT−/− ES) or puroΔTK are used. **c** Introducing pathogenic mutations into humanised alleles can be achieved by HR in zygotes using a ssODN (~150 bp) donor template combined with a locus-specific Cas9:sgRNA (no selection required). A similar strategy can be used for small-scale humanisation projects (small genes or partial humanisation) using a long ssODN (<2 kb) as a donor template and a pair of Cas9:sgRNAs. **d** Knock-in of large inserts (up to 200 kb) in both mouse and rat zygotes has been achieved by combining Cas9:sgRNAs and short ssODN donors with hybrid homology at the break-points between donor and target site to facilitate HR

As well as gaining biological insight, by analysing existing genomically humanised mice researchers are also gathering data for future genomic humanisation design and strategy—to guide decisions with potentially far reaching consequences on phenotype. Here, we first consider the biological, pathomechanistic and translational insights that have been gained from genomically humanised mice, and then we look at lessons learned so far which may influence future humanisation strategies. New technologies and resources (Box 1 and Fig. 1) are changing researchers’ abilities to genomically humanise the mouse, but they will only be successful if they are efficient, reliable, and reproducible and without significant off-target effects.

Box 1 Technology old and new Classical DNA targeting using plasmid vectors and homologous recombination (HR) in mouse embryonic stem (ES) cells has been the predominant technology to date to create genomically humanised knock-in mice (Fig. 1a), applied successfully to humanise loci < 15 kb (Tables 1 and 2), but no larger because of constraints on the insert size of plasmids used for targeting vectors. Among the earliest results were mice created to produce partially humanised antibodies by targeted humanisation of the mouse immunoglobulin kappa (light chain) and gamma-1 (heavy chain) constant regions^126,127^.

With the development of new vectors that carry tens of kb or more of insert DNA, such as bacterial artificial chromosomes (BACs), yeast artificial chromosome (YACs), human artificial chromosome (HACs) and mammalian artificial chromosomes (MACs), introducing much larger regions of human sequence became possible. BAC vectors combined with ES cell technologies have been used to humanise whole genes, gene clusters and even megabase-sized loci, via large-scale HR (Fig. 1a). Alternatively, recombinase enzymes and their short site-specific recombination sequences have been exploited for ‘recombinase-mediated cassette exchange’ (RMCE; Fig. 1b) and ‘recombinase-mediated genomic exchange’ (RMGE) strategies^55–58^.

While these methods have worked well, new CRISPR-based technologies for knocking-in exogenous DNA are changing the research landscape, albeit with potential off-target effects^130^. For example, CRISPR/Cas9-assisted HR in ES cells improves targeting efficiency, reducing the time and resources required to screen ES cell clones. This method was used to assess the optimal length of homology arms required for HR and to create a new strain of mice in which the *Kmtd2* (lysine (K)-specific methyltransferase 2D) gene was replaced by its ~ 42 kb genomic human orthologue (*KMTD2*), from the methionine start codon to the stop codon^128^.

Targeting with CRISPR/Cas9 is developing rapidly, and is increasing our capacity for genomic humanisation of rodent genomes. CRISPR/Cas9-assisted HR in zygotes, using a single-stranded oligodeoxynucleotide (ssODN) as a repair template (also known as ‘easiCRISPR’), is sufficient for efficient editing in zygotes without selection and therefore without genomic scarring. This has great potential for introducing pathogenic and other changes into large humanised regions, or for humanising small genomic regions: the current size limit for ssODN donors is ~ 2 kb, although this boundary is constantly challenged^131,132^ (Fig. 1c).

CRISPR/Cas9 combined with ssODN-mediated end joining is a striking new method for knocking-in long sequences directly to zygotes without the need to clone homology arms. Instead, a pair of short ssODNs harbouring short regions of homology, split between the donor template and target site, is used (Fig. 1d). This method was applied to knock-in 200 kb of human DNA including the *SIRPA* (signal-regulatory protein alpha) locus from a BAC vector, while removing the rat *Sirpa* homologous region, and in a separate experiment, to replace 58 kb of the rat *Cyp2d* (cytochrome P450, 2d) cluster with a 6.2 kb human *CYP2D6* gene^124^.

Finally, the mouse genome can also be genomically humanised by adding entire human chromosomes, with the purpose of modelling the complexities of human aneuploidy disorders, such as Down syndrome, which arises from trisomy of chromosome 21. This can be achieved by microcell-mediated chromosome transfer, whereby chromosomes in a human cell line are tagged with an antibiotic selectable marker; the cells are subsequently forced to multinucleate, which facilitates the isolation of individual chromosomes and the creation of ‘microcells’. These are fused with mouse ES cells, and used to generate mice via chimaera formation in blastocysts^54,60,61,102,129^.

## Insights from humanised mouse models

### Consider the question asked

It is possible to humanise individual base pairs and thus codons within a mouse gene to determine which amino acids are critical for the function or pathology under investigation. Experimenters can also choose a technically more challenging strategy of humanising entire exons, genes or even chromosomes, should the problem at hand not be addressable by smaller genomic alterations. Now that creating genomically humanised mice is becoming more straight forward (although still not routine), the questions these models are meant to address need to be clear a priori.

### Humanisation of specific amino acids

Individual codon(s) for a protein of interest can be humanised to determine whether the encoded amino acid has a critical impact on function. An early example comes from an investigation of the evolution of human communication: *Foxp2*^*hum*^ mice express a FOXP2 protein that has two ‘human’ amino acids that normally are not present in mouse FOXP2 (substitutions T302N and N324S). These residues are thought to have been positively selected during human evolution, possibly because of enhancing effects on speech and language^13^. *Foxp2*^*hum*^ mice have an intriguing gain-of-function phenotype, showing altered behavioural learning dynamics and increased corticostriatal synaptogenesis, which is opposite to the phenotype observed in *Foxp2* null mice. Thus, these finely humanised animals are helping to shed light on evolutionary adaptations of the human brain potentially required for the acquisition of speech capabilities^14,15^.

Another example of humanising specific codons to recreate human protein chemistry and to study the effects of mutation comes from research on Alzheimer’s disease (AD). In humans, the *APP* (amyloid precursor protein) gene, when mutated or present in three copies, can cause early-onset AD, which involves the deposition of the central Aβ peptide portion of the APP protein as extracellular plaques in the brain^16,17^. However, mouse APP does not form the typical amyloid plaques found in human AD. The reason appears to be that full-length mouse and human APP proteins differ by 17 amino acids, notably at three key residues within the central Aβ region that make the human protein more ‘aggregatable’ than that of the mouse^18^. AD researchers first humanised this Aβ domain in 1996 by targeting the three critical Aβ region codons, plus adding in a ‘Swedish’ familial AD mutation (causing two amino-acid substitutions immediately adjacent to the β-secretase site in APP), which resulted in enhanced Aβ production^19^. This came at a time when creating humanised knock-in animals was unusual and only 5 years after the first human *APP* mutations had been described in early-onset familial AD^16^. Subsequently, other AD researchers followed this same targeted humanisation approach for *APP*, but adding in other well-defined key familial AD mutations that affect Aβ processing within the rest of the protein^19–21^. This has resulted in extremely successful models of amyloid deposition and some of these mice are currently among the most widely used models in AD research, taken up by hundreds of labs worldwide^21^. Recently, the widely used ‘NL-F’ mice (carrying both the ‘Swedish’ (NL) and ‘Iberian’ (F) APP mutations) have been instrumental in showing that Aβ plaques can be seeded from cadaveric human pituitary growth hormone extracts into mice, and thus potentially also humans, highlighting the need to carefully assess the risks for Aβ seed contamination via medical procedures such as dura mater grafting^22^.

Fine-scale genomic humanisation of mouse genes can also be used to address the question of how different alleles of a protein of interest may play different roles in phenotypes. This is essential for understanding the biochemical interactions relevant to variation in human disease. To give an example, Apolipoprotein E (APOE) is a protein involved in lipoprotein binding and metabolism. Humans have three *APOE* alleles (-E2, -E3 and -E4) that differ at residues 112 and 158. The *APOE4* variant, which is associated with susceptibility to cardiovascular disease and AD risk, uniquely harbours Arg112 that causes another residue Arg61 to be exposed in the N-terminal domain enabling Arg61 to interact with the C- terminal domain (-E2 and -E3 variants neither have an exposed Arg61 nor such a domain interaction). However, while the single mouse APOE allele has APOE4-like Arg112, it lacks the human-specific Arg61 residue, lacks the domain interaction, and instead preferentially binds to high density lipoproteins, akin to the properties of human APOE3. When mouse APOE was humanised solely at residue 61 (Thr61 to Arg61) its lipoprotein binding preference changed to ﻿very low density lipoproteins (VLDLs) (akin to human APOE4), resulting in cognitive deficits and increased susceptibility to cardiovascular disease, consistent with human APOE4-dependent clinical features. In cardiovascular disease, increased binding of APOE4 to VLDLs leads to LDL receptor downregulation and increased LDL plasma levels (i.e., ‘bad cholesterol’ that can build up in the arteries)^23^; such an increase in LDL cholesterol is also a risk factor for dementia, which is thought to alter the deposition and/or clearance of Aβ, although the mechanism is not fully understood^24^. These key findings highlight that a specific domain interaction could be an important therapeutic target for ameliorating impairments in APOE4 in humans^25–27^.

### Humanising individual domains or exons

While introducing individual human amino acids can be critical for modelling human biology and pathology in mouse, this is not always sufficient. A dominant human pathogenic amino-acid mutation in the *VCP* (valosine containing protein) gene results in inclusion body myopathy associated with Paget disease of bone and frontotemporal dementia (IBMPFD) and ALS, but the same heterozygous mutation has little effect in mice^28^. For many models, the humanisation of specific stretches of amino acids or whole protein domains is key to answering mechanistic questions. One example that illustrates this comes from cancer research. The core DNA-binding domain of p53 (*TP53*, transformation related protein 53) is a hotspot for human cancer-associated mutations, and, thus, of outstanding interest for drilling into mechanisms of carcinogenesis. However, carcinogen-induced mutations in mice do not affect the same residues as in humans^29^. Therefore, to create mouse models suitable for understanding cellular processes in human cancer, key exons 4–9 of mouse *p53* (*Trp53*), which include the core DNA-binding domain, were humanised, resulting in the translation of a chimeric mouse:human p53 protein^30^. Subsequent introduction of human cancer-causing mutations gave new insights into how p53 mutations cause disruption to DNA damage-sensing and -response pathways^31^; and shed light on a human polymorphic p53 variant that may be associated with increased cancer risk in individuals of African descent^32^.

To study the effects of allelic variation on brain-derived neurotrophic factor (BDNF) function, a region of 274 bp of the human gene was inserted into the mouse *Bdnf* gene, including one of two allelic variants: Val66 or Met66^33^. Val66 is the major allele and also present in mouse, whereas Met66 in humans reduces BDNF activity-dependent secretion, leads to structural changes in the brain, and has been associated with psychiatric disorders and stress. Comparison of these mice showed that BDNF modulates competition in axonal branching^33^, which may be involved in Met66 allele-associated neurological dysfunction. Humanisation in this case, while providing a useful model to study human variation, was not critical since others have created a BDNF Met66 knock-in allele without humanisation to study anxiety-related phenotypes to good effect^34^.

Two allelic variants of the *OPRM1* gene have also been modelled by humanisation of the first coding exon of mouse *Oprm1*, including one of two variants: the major 118A allele and the minor 118G allele linked to alcoholism. The whole first exon was humanised because of the evolutionary divergence between mouse and human in this region, although the requirement for this extent of humanisation is untested. These humanised lines showed that the 118G allele enhances ﻿ventral striatal dopamine responses to alcohol, while also helping to refine translational studies by supporting the hypothesis that the 118G allele confers an enhanced response to the opiate antagonist drug naltrexone; a disputed link previously inferred from clinical studies^35,36^.

Continuing with the theme of preclinical translational research, monoclonal antibodies are an essential tool of modern medicine, but human and mouse epitopes often differ, which can be problematic for in vivo testing of human monoclonal antibody therapies in mouse. Humanisation of a mouse epitope can overcome this problem. For example, a single exon of the *Rankl* (*Tnfsf11*, tumour necrosis factor ligand superfamily, member 11) gene was humanised to recapitulate the region of human RANKL targeted by the monoclonal antibody drug Denosumab, which inhibits osteoclastogenesis and increases bone density; the resulting mice were important for preclinical testing of the efficacy of the drug (non-humanised mice do not respond) in treating specific bone disorders in humans^37^.

Partial humanisation can also be useful to recreate human-specific nonsense peptides that result from frameshift mutations that give rise to premature stop codons. For example, a splice-acceptor site mutation in the human *FUS* (Fused in sarcoma) gene that causes ALS leads to skipping of exon 14 and a frameshift in the coding sequence of exon 15 (resulting in a 14 amino-acid frameshifted C-terminus), followed by a premature stop codon. To recapitulate this mutation in the mouse, exon 15 of mouse *Fus* was humanised and the exon 14-skipping splice-acceptor site variant was introduced. Humanising only the splice site mutation would have resulted in a different and significantly longer frameshifted amino-acid sequence because of the differences in exon 15 between human and mouse. The partially humanised ‘FUS-Delta14’ mouse shows progressive motor neuron degeneration in a dominant, toxic gain-of-function manner and is currently one of the few physiological ALS models available. Importantly, the 14 amino-acid human-specific C-terminal frameshifted peptide has been instrumental in developing an antibody, which can be used to track the disease-causing protein in both mouse and human FUS-Delta14-expressing cells to help understand motor neuron death in ALS^8^.

Partial genomic humanisation of mice also includes the introduction of critical disease-causing non-coding DNA sequences such as introns and regulatory elements. In a mouse model of the recessive disorder familial dysautonomia, the humanisation of a disease-causing intronic sequence resulted in skipping of exon 20 of the *IKAP* transcript (IκB kinase complex-associated protein) in the central nervous system, resulting in a premature stop codon and protein depletion, which is also observed in patients. While protein depletion in the mouse was not sufficient to recapitulate neurological phenotypes of the disease, this model has been used for proof-of-principle studies for developing new therapies aimed at restoring functional protein levels^38^.

Thus, the humanisation of individual domains, regions and introns to create chimeric transcripts and proteins has provided the scientific community with highly informative mouse models for understanding basic mechanisms and test systems for translational research.

### Fully humanised genes and networks within the mouse context

The abundance of data from transgenic mice expressing human cDNAs, or genomic sequences from plasmid or BAC vectors, reassures that generally mouse and human proteins carry out equivalent functions, and that fundamental processes such as the recognition of splice boundaries are conserved between the two species. The relatively few examples of full genomic humanisation of mouse genes—i.e., the replacement of the entire protein coding region, including introns, with the human sequence—generally confirm that the human genes can successfully replace their mouse orthologues, albeit with sometimes minor changes in gene expression.

Fully genomically humanised mice have been created to recapitulate human biology and to achieve the greatest physiological relevance in disease modelling or therapeutic development, rather than to address questions of normal protein function. For example, the mouse *Apoe* gene discussed above has been fully genomically humanised in order to gain further insight into the role of APOE in disease, including the effects of the individual alleles. All three human *APOE* polymorphic variants (*APOE2*, *-E3* and *-E4*) were knocked-in to the mouse locus (all three coding exons plus intervening introns), placing them under the control of the mouse promoter. These models have been widely used to better understand the molecular basis for atherosclerosis and AD while also providing a means to test therapeutics in a humanised context^39–42^. We note that such models may be crossed in classical genetics approaches to provide insights into human biochemical pathways. Humanised *Apoe* mice, for example, have been crossed with Tau transgenic mice, which revealed that APOE4 aggravates tauopathy independent of ﻿amyloid-β pathology^43^.

Under certain circumstances, it may be necessary to humanise multiple genes within a pathway or network to better model human biology. Chimeric mice harbouring a human immune system can be generated by engrafting immunodeficient mice with human haematopoietic stem cells; however, mouse cytokines poorly cross react with human receptors and are inadequate to support the maturation and function of these human immune cells. Cytokines can be administered to the mice to overcome this problem, but to achieve physiological expression levels and distribution, several cytokine genes have now been fully genomically humanised in mouse, and successfully support the human cellular component of chimeric animals. Used in combination, these knocked-in cytokine genes help improve the development and function of human monocytes, macrophages, and NK cells in the mouse environment^44–48^.

Moving onto translational research, a novel method for production of high affinity monoclonal antibodies against human Complement C5a Receptor 1 (C5AR1), a therapeutic target for various inflammatory conditions, was enabled by humanising the complete mouse *C5ar1* gene^49^, using neutrophils from C5AR1 humanised mice (expressing high levels of human C5AR1) to immunise wild-type mice. C5AR1 humanised mice were subsequently successfully used for preclinical testing of the antibodies. For pharmacokinetics analysis, a number of xenobiotic sensors and drug metabolising enzymes have been fully humanised to help predict human drug responses^50–53^.

Although there are relatively few published examples in which entire mouse genes have been replaced with the human gene, these genomically humanised animals are valid models for understanding human biology and pathology, and have provided tools and given insight that would not have been possible with studying the mouse orthologues alone.

### Genome humanisation for investigating complex disease loci

The largest region of humanisation within the mouse genome extends over tens of megabases of DNA within an almost complete human chromosome 21 (Hsa21)^54^. In mice developed to produce human antibodies, humanised regions are several megabases in length^55–57^. In one of the first large-scale genomic humanisation studies of gene expression, the humanised region was almost 200 kb long, located in the *α globin* gene cluster^58^. In all these cases, perhaps surprisingly, phenotypes ranged from relatively mild to apparently no different from wild-type mice (other than for the production of human proteins). The largest published region of humanisation currently comes from studying the complex human chromosomal disorder Down syndrome, which is an aneuploidy syndrome resulting from trisomy of Hsa21. Modelling this disorder in mice poses a problem because the content of Hsa21 is distributed over three syntenic regions on mouse chromosomes 10, 16 and 17^59^. Thus, transchromosomic mice (‘Tc1’ and others) were created via microcell-mediated chromosome transfer of Hsa21 into mouse embryonic stem (ES) cells and subsequent production of chimeric mice via blastocyst injection, followed by breeding of chimeras for germline transmission of the human chromosome^54,60,61^. Tc1 mice, which carry an extra > 42 Mb of human DNA, recapitulate many features of Down syndrome, most of which are fairly mild (on the genetic backgrounds studied) and include neurological, cardiac and mandible phenotypes^54^. However, Tc1 mice lose the human chromosome stochastically, more from rapidly dividing cells such as those in the thymus, than, say those in the brain (where ~ 67% of cells retain the human chromosome), and the resulting mosaicism probably contributes to the mildness of the phenotypes^54^.

Efficient production of human monoclonal antibodies from human immunoglobulin IgG light and heavy chain knock-in mice has been achieved, by two different groups independently, by megabase-sized knock-in of the variable regions of both heavy and light chain human IgG loci^55–57^. Both groups implemented iterative targeting of kilobase-sized segments in ES cells using BAC vectors; one group used a homologous recombination (HR) strategy, while the other utilised recombinase-mediated cassette exchange (RMCE) alongside the PiggyBac transposase system for megabase-sized scarless editing. Remarkably, in both approaches, these knock-in mice show no functional impairments in their immune systems, which is in contrast to previous non-targeted humanisation efforts. They have been used for testing therapies against HIV-1 (human immunodeficiency virus-1) and developing human therapeutic antibodies to treat diabetes^55–57,62,63^. More recently, an alternative one-step approach was devised to knock-in pre-arranged human heavy chain sequences (~ 1.9 kb; directly into zygotes) for generation of antibodies against critical HIV-1 epitopes^64^.

Genomic humanisation of large complex loci has shed light on important non-coding regulatory sequences, which would have been difficult in conventional mouse models. Humanisation of the *α globin* gene cluster, via recombinase-mediated genomic exchange (RMGE), was undertaken to study gene expression from this clinically important region and represented a proof-of-principle that humanising large genomic loci is possible. One outcome of this research was the finding that globin gene expression levels differ between the humanised mice and wild-type animals. Further dissection of the underlying cause showed that the HS-40 element in this cluster was critical for *α globin* gene expression within the human, but not the mouse locus^58,65^.

The surprising conclusion from reports of mice in which large regions of the genome have been replaced or human sequences added, is that phenotypes are generally mild without an apparent effect on mouse welfare. This is at least true for wild-type loci. However, even in the largest genomic humanisation effort reported to date, the Tc1 mouse model of Down syndrome, <1% of the genome is human DNA.

## Lessons learned so far for humanisation strategies

**Fig. 2:**
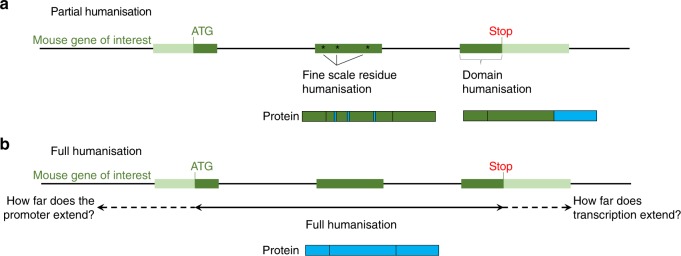
How far to humanise. A summary of considerations when deciding on the extent of targeted genomic humanisation for a given gene of interest. Dark green boxes represent exons, lines between exons are introns, light green boxes are UTRs and blue regions represent humanisation. **a** Partial humanisation typically involves humanising specific residues and/or domains of interest. Fine-scale humanisation of specific amino-acid residues can be performed in isolation if they have known biochemical differences from mouse to human, or if only a small number of residues need to be altered to achieve a human protein sequence. Specific domains or exons can be humanised if, for example, they are known to be critical for human disease. Examples of translated protein products are given. **b** Full humanisation involves humanising the whole gene, including introns, to attain translation of the full human protein, potentially including human-specific splicing patterns, and for maximum translational potential. 5ʹ and 3ʹ-UTRs, promoters, and other regulatory sequences can be included, on a case-by-case basis, if understanding of gene regulation is the question at hand, if gene clusters are to be humanised or if pathogenic mutations fall within such flanking regions

The rules of the genome are not fully understood, nor are the full phenotypic consequences of creating mice with human genomic DNA. However, although genomically humanised mice are relatively few, it is clear that they can give insight into biology and pathology that cannot necessarily be gained from conventional mouse models. Advances in genome engineering technologies, including CRISPR (clustered regularly interspaced short palindromic repeats (CRISPR)-Cas approaches (Box 1), mean that making humanised models is easier and faster than ever before—although still not a conventional route—such that even when the specifics of mouse:human transcript or protein differences are unknown, humanisation should be considered. However, the design of a humanisation strategy is far from routine, and a key question remains: how far to humanise (summarised in Fig. 2)?

### To keep things simple or go full scale?

The decision whether to humanise just a codon, just a domain, just the coding region, or to go full scale and include introns and flanking regulatory regions depends on the questions being asked, as we have discussed above. Most genomically humanised mice in the literature have fine-scale changes within the coding sequence that result in chimeric human:mouse proteins, facilitating the study of critical aspects of protein biochemistry, without greatly altering mouse genome architecture.

To work with models that contain full-length human genes, perhaps in trying to produce human-specific splice patterns and regulation from intronic sequences, human genomic architecture has to be introduced. Mice have on average of ~ 2.4 splice isoforms per gene, whereas humans produce ~ 3.4 isoforms per gene^66^ and individual splice isoforms can be disease causative. For example, the isoform ratios of TAU, encoded by *MAPT*, are critical in causing frontotemporal dementia, and while a genomically humanised *MAPT* mouse has not yet been described, encouragingly a human BAC transgenic recapitulates human *MAPT* splicing in mouse^67^. Thus, if the full repertoire of human splice isoforms is required, then full-scale humanisation across the gene of interest could be attempted.

Exclusion of introns, which might help to keep things simple in a humanisation strategy, can potentially be disruptive. For example, when the xenobiotic sensor *PXR* (Pregnane X receptor) was originally humanised, some introns were excluded—it was later discovered that this caused a mis-splicing event as a result of exons 7 and 8 fusion, leading to creation of a new splice-acceptor site and variant whereby exon 5 spliced to exon 8. The gene was subsequently fully humanised to eliminate this variant^52,53^. Thus, and perhaps not surprisingly, in at least some cases, maintaining the human genome architecture is likely critical for normal gene expression in mouse.

We note that in moving towards translational research, the presence of the full-length human transcripts and proteins maximises the potential to test therapeutics relevant for use in humans. An excellent example of this comes from the need to determine the optimal dose of human antisense oligomer (ASO) therapies against the human rather than mouse mRNA sequences, because in the clinic the oligomer must bind to the human sequence; as, for example, in ASOs that modulate APOE levels^42^.

### Considering flanking regulatory sequences

If an investigation warrants the genomic humanisation of an entire mouse gene, which is coding regions and internal introns, the decision over how far to extend into the 5ʹ and 3ʹ regions currently relies largely on a combination of limited information, expediency, and guesswork. It is essential to look closely at how far the gene of interest extends upstream and downstream, as apart from different transcripts, the gene may in addition produce alternative 5ʹ and 3ʹ exons that contain different untranslated regions (UTRs), which may affect gene regulation. However, even with this information, generally too little is known about the regulatory sequences within most genes to judge whether to humanise only from the first ATG to the last stop codon or to humanise from the start of the first 5ʹ UTR (if known) to the end of the last 3ʹ UTR (if known) of alternative transcripts. For single genes, a conservative approach is to humanise between outermost known start and stop codons only. A riskier approach—but one that may result in more faithfully recapitulated human gene regulation—is to include the human UTRs. However, currently there is insufficient information to reliably predict the outcome of this strategy and to make generalisable rules.

A critically important feature for gene regulation is the gene promotor, but currently no good spatial definition of the promotors of most genes is available. Often the best that researchers can do is to assume that the CG-rich region upstream of a gene includes the promotor, and to look carefully for transcription factor binding sites within or nearby this genomic sequence. In fact, so far targeted humanisation of most single genes has not included promoter sequences, just in case critical promoter–enhancer interactions were to be disrupted. Furthermore, some regulatory elements may control genes long distances away^68^, for example, in human and mouse the 1Mb-distal ZRS enhancer for *Sonic hedgehog* (*Shh)* is critical to limb-specific *Shh* expression^69^.

One potential limitation of genomic humanisation strategies that include the promotor regions is that even though human and mouse transcription factors are conserved, orthologues do not necessarily have identical amino-acid sequences or recognise identical motifs. Therefore, mouse proteins might not correctly regulate the transcription of human genes. This problem was addressed to some extent by the Tc1 mouse model of Down syndrome that carries Hsa21, mentioned above. The researchers investigating transcription factor binding found that mouse transcription factors did indeed bind to the human cognate sites, despite the sequences often being slightly different from the mouse orthologous sites. This result may allay some fears about introducing human promoters for human genes into the mouse cellular environment^70^. This result was in part corroborated by a recent RNA deep sequencing study of Hsa21 in both the Tc1 mouse and in human cells;^71^ specifically, mouse *trans* acting factors (such as transcription factors) appeared responsible for regulating humanised gene expression. This was evident since humanised gene expression levels in mouse closely correlated with levels of the mouse orthologous gene, and not the human gene in human cells^71^, in contrast to findings of the earlier study that suggested human expression levels were maintained^70^. Importantly for humanisation studies, splicing patterns of genes on the humanised chromosome in Tc1 mice closely recapitulated splicing patterns in human cells, even in human non-coding genes that do not have mouse orthologues^71^, indicating that regulation of splicing is intrinsically sequence dependent and that splicing machinery is well conserved between mouse and human.

Finally, expediency comes into play in targeting strategies when humanising a sequence might disrupt important mouse features. In a project we have underway, we find that the 3ʹ-UTR of a mouse gene of interest overlaps the 5ʹ-UTR of the adjacent gene, whereas in the human genome the two orthologues are separated by several kb. In this case, our humanisation strategy extends only to the stop codon of the gene of interest, and no further, to ensure that the mouse 3ʹ-UTR is not disrupted, and we do not inadvertently alter the regulation of the downstream gene.

### Genomic humanisation of sequences that do not exist in mouse

Approximately 1% of human genes do not have a mouse orthologue and vice versa^72^, and some of the 99% of homologous protein coding genes may exist in different copy numbers in the two species. For example, mice have a single *Smn* gene encoding the survival motor neuron protein, but humans have *SMN1* and up to four copies of its paralogue *SMN2*. *SMN1* and *SMN2* are almost identical but a single-nucleotide change in exon 7 of SMN2 results in ~80% of its transcripts producing an unstable, truncated protein and only ~20% of *SMN2* mRNA encoding functional protein. This becomes critically important when studying type 1 spinal muscular atrophy (SMA), a recessive disease with an incidence of ~1 in 10,000 depending on the population, which arises from mutation in *SMN1*. Left untreated, children with SMA die before 4 years of age from respiratory failure due to lower motor neuron death^73^. This form of SMA has been modelled by strategies that include targeting human *SMN2* into *Smn* null mice, for example, to recapitulate the human condition (ref. ^74^ and papers therein).

We wish to raise for consideration the following (so far hypothetical, but possible) scenario: where human paralogs have taken on different functions, but only one mouse homologue exists, humanising the mouse gene may potentially only produce a protein that functions as a single human paralog, leaving the remaining functions of the other human paralog(s) absent from the mouse.

Triplet (and larger) repeat expansion diseases have not been found in mice, and yet are a well-known cause of neuromuscular, neurodegenerative and other disorders in humans. Genomic humanisation is a route to model these disorders in mice; for example, in humans, a CAG expansion in exon 1 of the androgen receptor (*AR*) gene results in a polyglutamine (polyQ) tract in the protein and can lead to spinal bulbar muscular atrophy (SBMA). Mouse *Ar* exon 1 has significant sequence divergence from the human orthologue and so a model was created that humanised 1340 bp of exon 1, including a human-specific 113-residue polyQ-tract. This has proven successful in uncovering disease mechanisms and developing therapeutics for SBMA^75–82^.

Huntington disease (HD) is another polyQ-expansion neurodegenerative disease, which has been modelled extensively via BAC transgenesis (producing fast, neurodegenerative phenotypes) and via knock-in strategies (slowly progressive phenotypes). Humanised knock-in HD models consist of humanisation of exon 1 of the mouse *Huntington* (*Htt*) gene, which includes a pathogenic polyQ-tract adjacent to a polypurine tract^83–87^ (both of these features show poor conservation between mouse and human). Such mice are available as an allelic series derived from natural expansion of the CAG repeat in vivo, ranging from those with mild molecular changes to those with progressive behavioural and motor dysfunction. Non-humanised knock-in HD mice (expanding the mouse polyQ tract alone) have also been generated with similar phenotypes; nevertheless, humanised models have been widely used to gain insights into disease pathophysiology, including characterising the timing of early neuropathological changes, identifying posttranslational modifications crucial to mutant *HTT* pathology, and omics profiling to understand repeat length-dependent dysfunctions^88–91^. Humanisation has also allowed successful therapeutic testing of gene editing strategies targeting human exon 1 with the aim of excising the repeat expansion^92^.

When the genomic context of a disease-causing mutation is entirely specific to humans, humanisation is the only option. For example, an SVA retrovirus insertion into the 3ʹ-UTR (located in exon 10) of the human *FKTN* (FUKUTIN) gene causes Fukuyama muscular dystrophy^93^. SVA retrotransposons are hominid specific and do not exist in mouse. Therefore, the whole of mouse *Fktn* exon 10 was humanised, with and without the SVA insertion, to replicate any sequence-specific effects the insertion may have on the host gene. Indeed, this turned out to be important as the SVA insertion caused an abnormal 3ʹ splicing pattern in both the mouse model and human patients—leading to severely reduced protein expression. The humanised model mimics some—but not all—of the human disorder phenotype, and has been useful for understanding disease mechanism. This example demonstrates that introducing a wider genomic context to the disease-causing mutation can be essential for replicating pathogenic mechanisms^93^.

In genetically complex disorders, differences between mouse and human might be so prominent—both in genomic context and biology—that a considerable investment in understanding the locus or loci of interest has to be made before a detailed approach to modelling can be developed. β-Thalassaemia in humans results from mutations in the *β globin* locus that lead to a reduction of functional adult β-globin protein levels. Simple replacement of the two mouse genes for adult *β globin* with the single human *β globin* gene containing an intronic causative β-thalassaemia βIVS-2-654 mutation led to classic signs of β-thalassaemia intermedia in heterozygous mice^94^. These mice have been used to test therapies that either block (using ﻿splice-switching oligonucleotides) or correct (via gene editing) the cryptic splice site introduced by the mutation^95,96^. However, divergence in sequence (four genes in mouse, five in human) and timing of embryonic-to-foetal-to-adult *β-globin* expression between mouse and human complicates modelling of both β-thalassaemia major and sickle β-thalassaemia—severe, recessive forms of anaemia characterised by a lack of functional adult β-globin. Human patients survive untreated for some time postnatally, supported by foetal β-globins, which do not fully switch to adult β-globins until 1 year of age. Simply knocking-out mouse adult β-globins fails to model these recessive anaemias because mice lack a bona fide foetal haemoglobin and the knock-out mice are non-viable because mouse foetal liver erythropoiesis depends on adult β-globins. Thus, to model human recessive thalassaemias, a bespoke humanised approach was required that involved the replacement of the mouse adult *β globin* genes with a ﻿delayed foetal-to-adult haemoglobin-switching transgene enabling survival to birth^97,98^. Postnatal survival was later further improved by introducing a ‘hereditary persistence of foetal haemoglobin’ mutation within the human γ-*globin* gene component^99,100^. Thus, gaining a detailed understanding of the architectural and expression differences between mouse and human loci may be necessary to inform how best to introduce human sequences into a mouse genome to obtain the most informative results.

### Humanising entire chromosomes

The genome contains features beyond the primary genetic sequence that may affect humanisation strategies. A possible example of this arises in the Tc1 mouse model of Down syndrome, which stochastically loses Hsa21 over time, resulting in mosaic mice^54^. This may be due to the centrosomes of the mouse cells not properly recognising or interacting with the centromere of the human chromosome. New types of human artificial chromosomes (HACs), including those with mouse centromeric sequences, are likely to give greater stability to human chromosome-sized regions in mouse^101^. Similar to the Tc1 model, microcell-mediated chromosome transfer was employed to create mice harbouring human minichromosomes carrying the entire immunoglobulin heavy and kappa light chain loci, combined with knock-out of the orthologous mouse loci, to successfully express fully human antibodies^102^. Of note in this case, minichromosomes were found to be mitotically and meiotically unstable and mice had impaired B-cell development and partial immune deficiencies.

### Full genomic humanisation is not always the best strategy

Finally, it may be beneficial not to humanise specific domains when they, for example, have important functions in determining cellular localisation. In humanisation of both *Prnp* (Prion protein) and *Ctla4* (cytotoxic T lymphocyte-associated 4), the mouse signal peptide sequence in each of these genes was retained, to avoid potential problems with translocation to the cell membrane; since signal peptides are cleaved, this should have no effect of the biochemistry of the mature protein^103,104^.

### Unexpected effects

The phenotype of a genetic mouse model—transgenic or genomically humanised—is not always predictable, but when unexpected outcomes arise, such models can provide valuable insight from studying the mechanism of why the observed outcome is different from expectation. As an example, to model myotonic dystrophy, the 3ʹ end of the *Dmpk* (dystrophia myotonica protein kinase) gene was humanised, including the addition of 84 CTG repeats in the 3ʹ-UTR, which leads to pathology in humans. In *Dmpk*-humanised mice, however, this repeat number failed to produce a pathogenic phenotype. Nevertheless, this model gave important insight into the somatic instability of the repeats, and provided evidence that repeat stability is associated with ﻿the activity of mismatch–repair (MMR) machinery^105^.

Similarly, in larger-scale humanising of the introns and exons of a gene, the mouse environment (chromosomal or cellular) may cause unexpected phenotypes. Currently, this is unpredictable and must be assessed on a case-by-case basis, but does not preclude working with such animals. For example, the large-scale humanisation of the α globin locus (knocking-in 117 kb of human DNA, deleting 85 kb of syntenic mouse DNA) resulted in significantly reduced expression (40% of mouse α globin expression levels) of α globin from the humanised locus, but the developmental pattern of α globin expression was retained and histological markers were normal, allowing evaluation of the mechanistic outcomes of point and indel mutations at this locus^58^.

Genome architecture can be disrupted by scars in the genomic landscape (i.e., introduction of exogenous sequences that could, for example, disrupt promoters or enhancers and affect gene regulation). An experiment to humanise rhodopsin included fusion of green fluorescent protein (GFP) at the C-terminal end, in order to visualise Rhodopsin-expressing cells in the retina with high sensitivity; however, the GFP fusion generated a recessive allele, unexpectedly causing ﻿death of rod photoreceptor cells, thereby providing a useful, even though unintended, model for retinal neurodegeneration^106^. In the same study, other humanised alleles were generated that also included the presence of recombinase recognition lox sites flanking Rhodopsin–GFP, which significantly reduced translation of the gene, and further exacerbated retinal degeneration. Thus, introduction of exogenous sequences into a locus should be carefully considered and evaluated for unintended effects. Certain forms of genomic scarring, such as frt sites left behind from exogenously inserted selection cassettes (needed during genome engineering in ES cells; see Fig. 1a), can now readily be avoided by using the PiggyBac transposase system or CRISPR/Cas9 targeting in zygotes (which does not require selection, and therefore leaves no genomic scar) (Fig. 1b, c).

Another unexpected, but informative, outcome of humanisation comes from the Tc1 mouse model, which was produced to understand Down syndrome phenotypes, but in fact also gave insight into the expression of primate-specific repeats. Over 50% of the human genome is derived from repetitive elements, mostly from transposable elements that can act as regulatory DNA and whose activity is controlled by several different mechanisms. Placing Hsa21 into the mouse nuclear environment resulted in transcriptional activation of transposon-derived human regulatory regions, which could potentially influence gene expression of nearby transcripts on this chromosome; among other results, this highlighted the latent regulatory potential of human-specific elements^107^.

Importantly, we also wish to highlight the need to work with a wild-type genomically humanised animal as a control, where possible, when studying genomically humanised mutants. This is to ensure that unexpected phenotypes that derive from the human DNA per se are distinguished from those associated with the mutation, in particular when introducing many kb- or Mb-sized stretches of DNA.

### When far is too far

Just as important as the technical and strategic considerations regarding genomic humanisation, are the ethical issues—how far can humanising the mouse genome go while still having a mouse? This is an unresolved question that was discussed in a report by the UK Academy of Medical Sciences (‘Animals containing human material’, 2011), which at the time cautiously concluded there were no new ethical challenges. However, since then the field of genome editing has exploded with new technologies and these considerations may have to be revisited in the near future. This issue is of course also pertinent to the mouse:human cellular chimeras that have been made for many years with the purpose of studying the immune system and which are now also being created, for example, to better understand brain function and dysfunction^108,109^.

## Genomic humanisation going forward

The technology for targeted genomic humanisation of the mouse requires further development to streamline approaches and to simplify working with long and challenging sequences such as expanded repeats. In that regard, CRISPR/Cas systems are changing rapidly, offering great potential for genomic humanisation that is only just beginning to be realised. Modified Cas9 variants, including base editors and variants with alternative protospacer adjacent motif specificity, now permit more precise editing than ever before^110,111^, whereas structural or copy number variants can be modelled in mice following humanisation of any given gene^112^.

Nevertheless, many technical challenges remain. For example, several important human disease genes are now known to undergo repeat-associated non-ATG (RAN) translation—often producing peptides in all three or even all six reading frames (when taking into account translation in the reverse direction)^113^. Producing humanised mouse models that carry stable repeats to understand associated pathomechanisms is not yet routine. Furthermore, for at least some of these repeats the flanking sequence contexts may be important for disease manifestation^114^, meaning that, in addition, the entire human gene sequence has to be knocked-in as a control.

Even in cases of relatively straight forward humanisation of a single wild-type gene, as described in the examples above, it is not always clear how far one should extend genomic humanisation in order to most faithfully recapitulate human gene expression in a mouse context. In eukaryotes, for example, it largely remains to be determined where and how transcription terminates, which may be many bp or even kb beyond the polyadenylation signal in a transcript. Transcription terminator proteins are involved in releasing mRNAs and RNA Polymerase II from each other^115,116^, and this is coupled to cleavage and polyadenylation of the 3ʹ end of mRNAs and associated with the effects of transcription boundary-associated RNAs, which include terminus-associated RNAs^117^. Termination can occur at different sites 3ʹ of the polyadenylation signal, and usually it is not known how far to humanise beyond the 3ʹ-UTR of a gene. Yet, these sequences can have profound effects on transcription and translation^118^.

Similarly, codon usage is clearly important for translation, affecting, for example, mRNA stability, ribosome binding, mRNA processivity, and other phenomena^119^. However, as mouse and human are only separated by 75 million years of evolution, it is assumed that codon usage will have similar effects in both species, but this may not be the case for specific genes.

Thus, the optimal strategy for genomic humanisation is likely to be only discernible on a gene-by-gene basis following considerable prior investment in gathering data on human gene expression in human cells and tissues. However, existing models clearly show the utility of working with current knowledge, and have in fact helped to find new regulatory sequences that would have been hard to find otherwise^2,58,65^.

In making genomically humanised mice, unexpected insights about coding regions can be gained; for example, for some pathogenic human alleles, such as the A53T mutation in *SNCA* that causes Parkinson’s disease, the human disease variant is the wild-type sequence in the mouse. This well-known phenomenon occurs at least in some cases because of compensatory evolutionary changes at specific sites elsewhere in the gene/protein^120,121^. Thus, humanisation of the complete gene, with the normal human sequence (aberrant in mouse) and the disease human allele (wild-type in mouse) may provide a route to studying phenotype. Alternatively, the evolutionary compensation of variant residues could occur within other genes that interact with *SNCA*, meaning that multiple genes might have to be humanised to yield a phenotype of interest.

In modelling human disease in an age of personalised medicine, humanisation of a single allele will not be sufficient to address the great variability between individuals, including response to treatment and clinical trajectories. This remains a challenge for mouse modelling in general, not just for creating genomically humanised models. Furthermore, humanisation of a single gene may not be sufficient to understand biology or pathology—particularly when protein complexes or ligand–receptor interactions are involved. Thus, at a minimum, the between-species conservation of these interactions should be considered. Future approaches to humanised mouse models may lie in humanising entire networks or pathways.

Genome wide association studies have identified thousands of disease risk loci in the human genome, the majority of which lie in non-coding regions. Modelling non-coding variants in animals is challenging, due to the lack of evolutionary sequence conservation in many non-coding regions. Recently, liver-associated expression quantitative trait loci (eQTL) were identified via screening in hepatocyte cell lines derived from a cohort of healthy volunteers^122^, as part of a study to identify lipid-functional genes and variants, and to better understand human complex traits. In the same study, one of the lead eQTLs (rs2277862; T) was modelled in mice via CRISPR-Cas9 mutagenesis in zygotes, including humanisation of four surrounding nucleotides such that the wider region became identical to the human sequence, leading to significant reduction in expression of the nearby *CPNE1* gene, whose function is still to be determined. Thus, technological advances in genome engineering combined with the ever improving understanding of genomic architecture render genetic modelling of non-coding variants an exciting future prospect^123^.

The development of CRISPR/Cas technologies means that the mouse is no longer exceptional and in fact data on genomically humanised rats and pigs are already emerging^124,125^. Future humanisation strategies may thus involve a range of model organisms, which inevitably means new ethical boundaries will have to be defined. Finally, to facilitate a reduction in the use of animals for research purposes, humanised mouse models can and should be studied in parallel with human tissue samples and human in vitro models, which will at the same time further help in obtaining a more complete picture of human biology and pathology.
